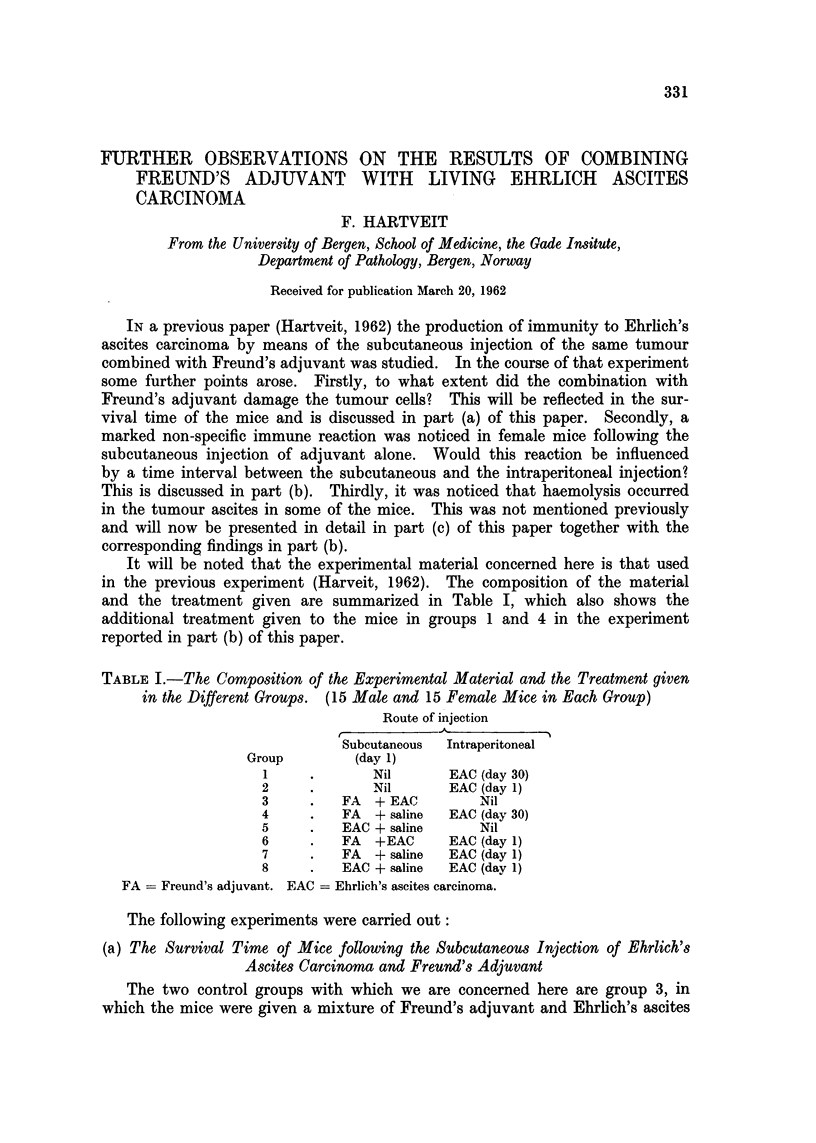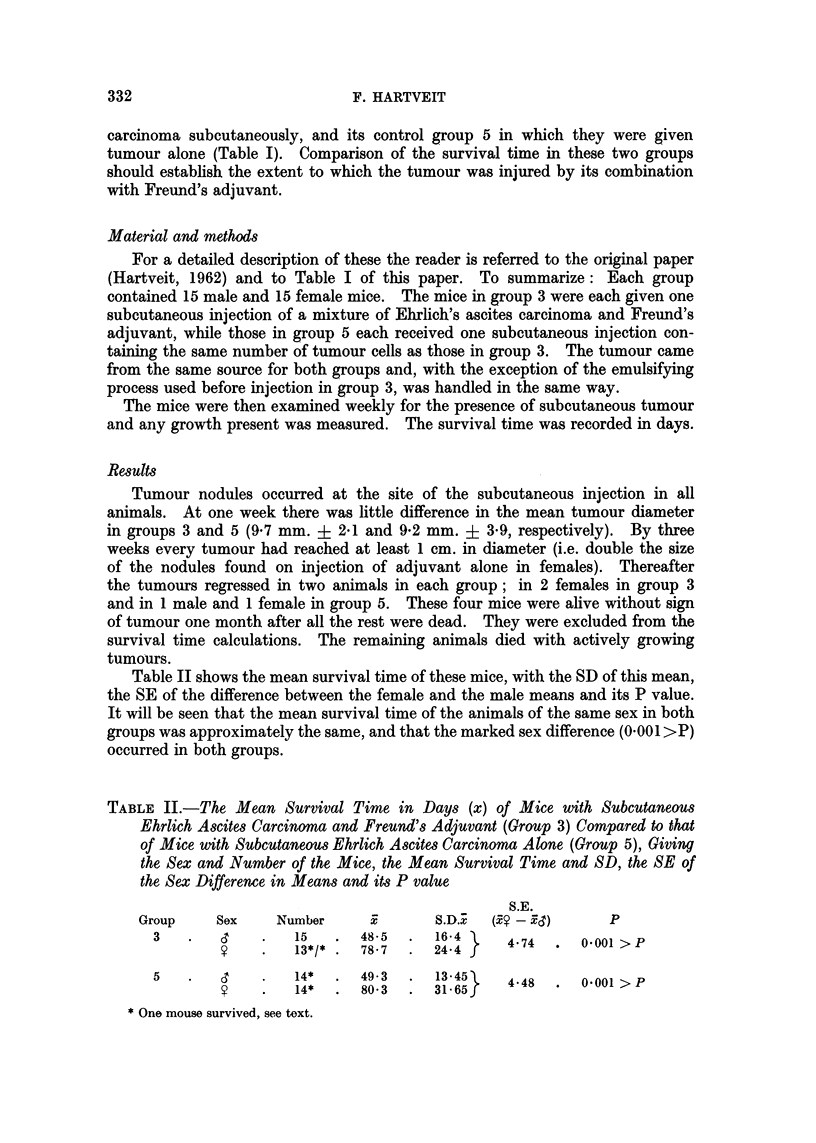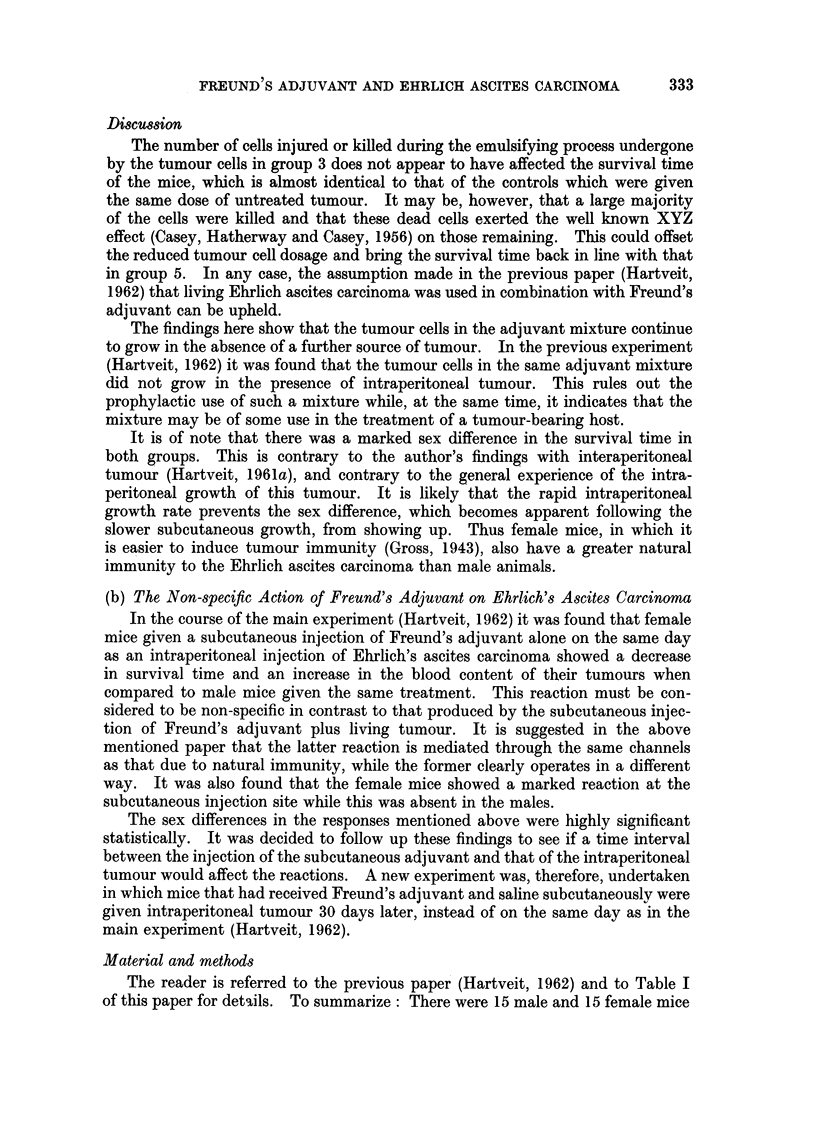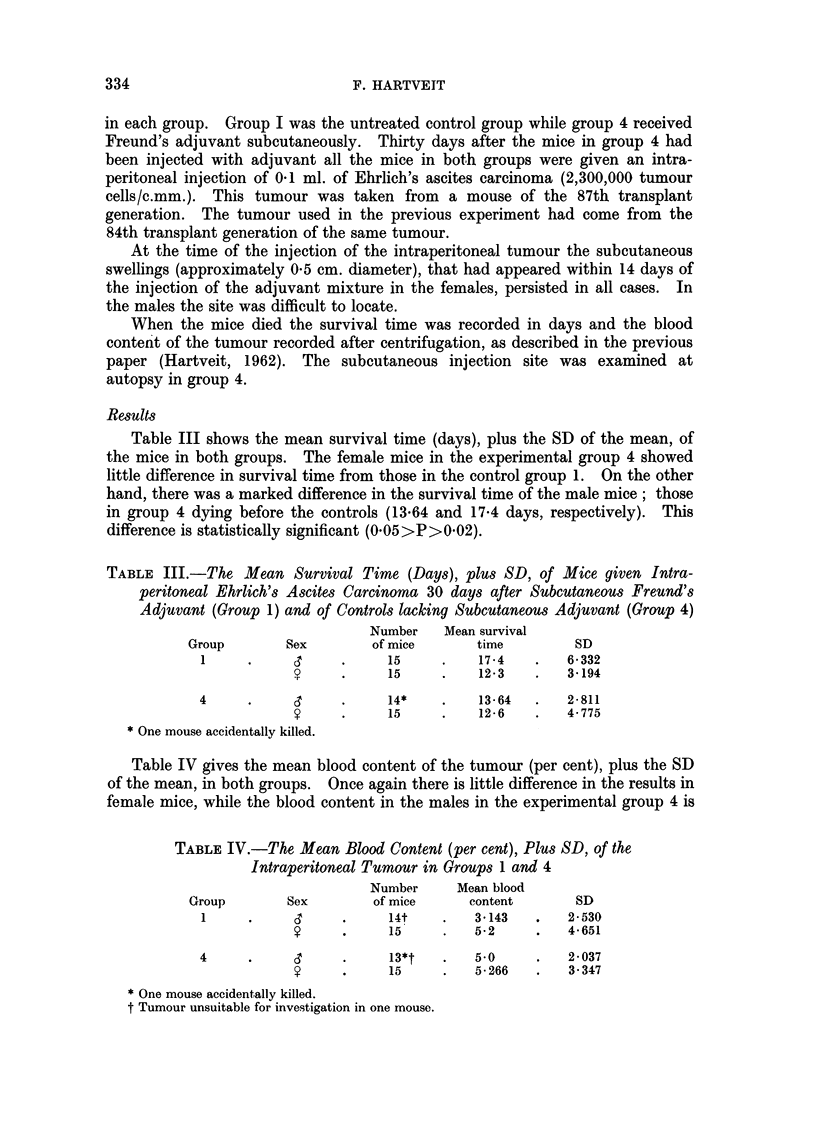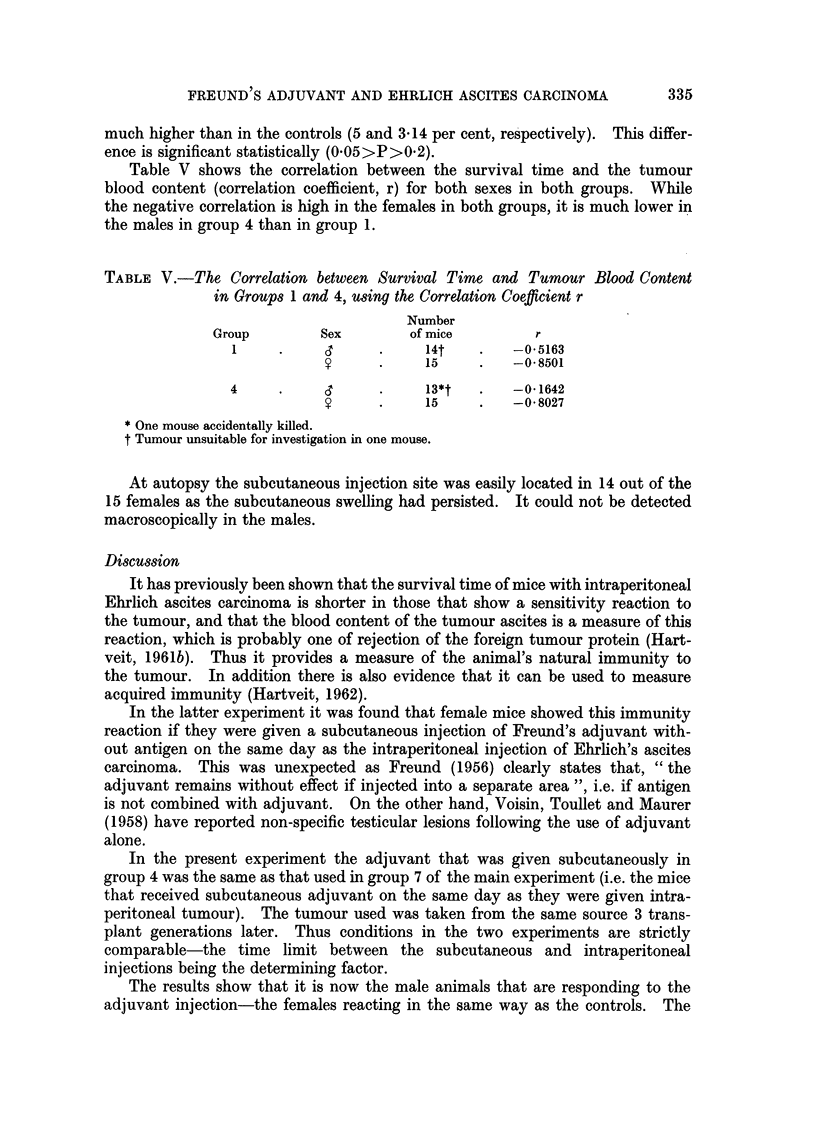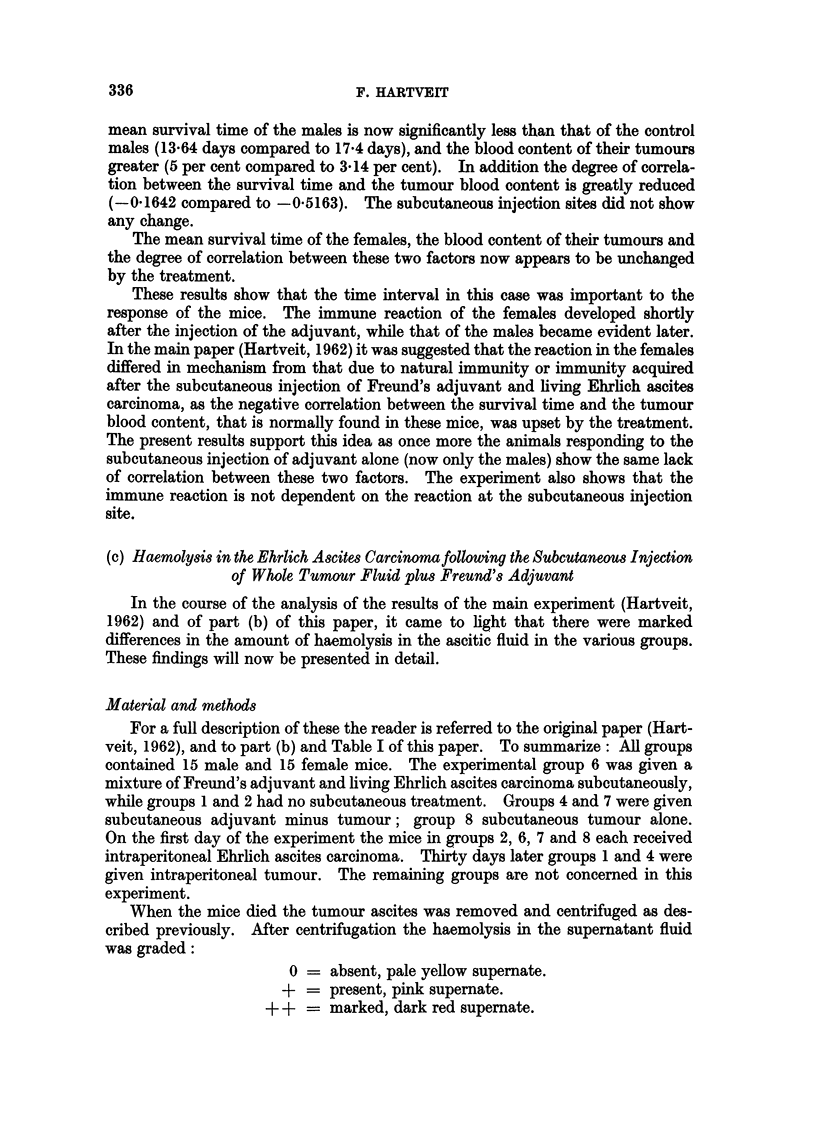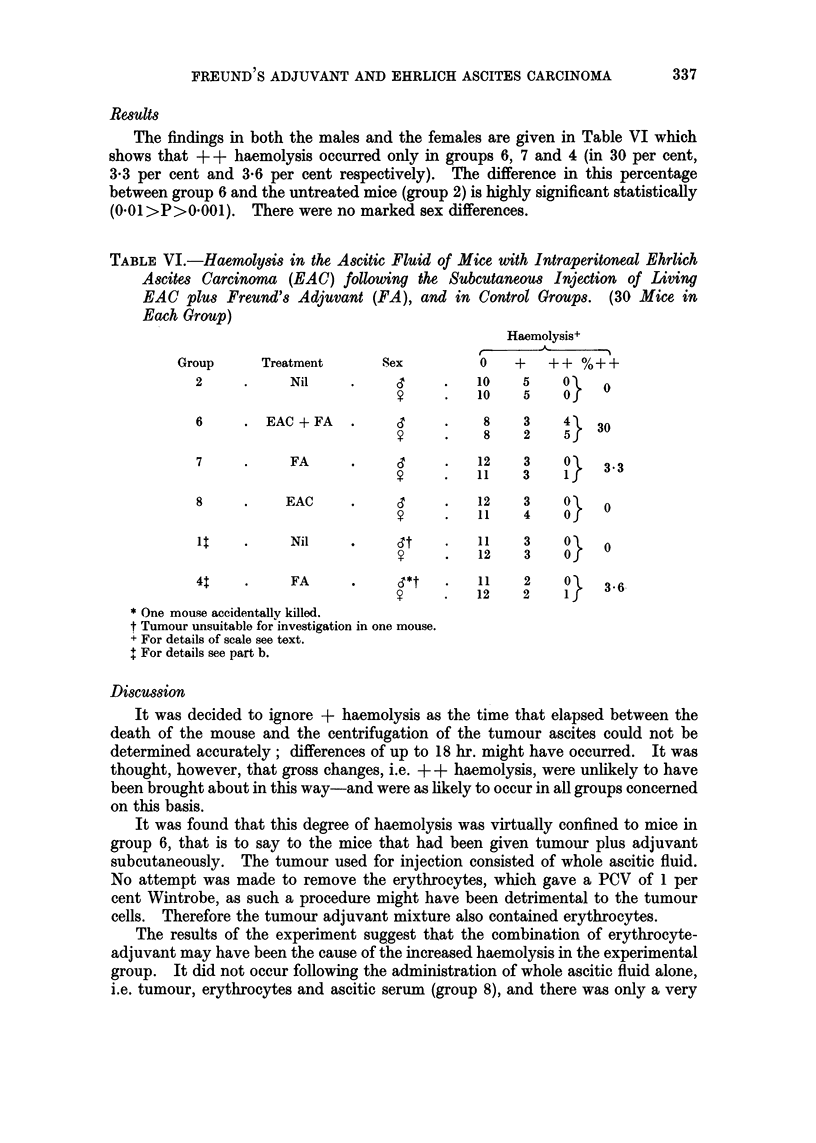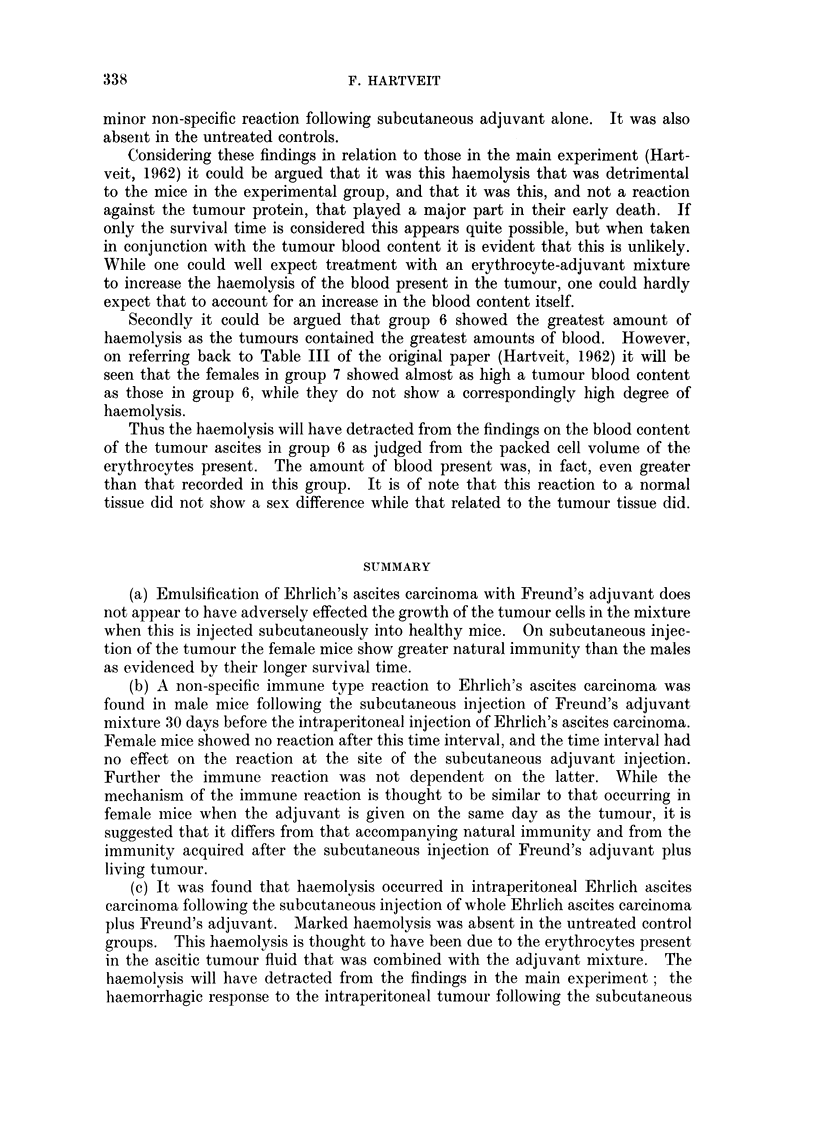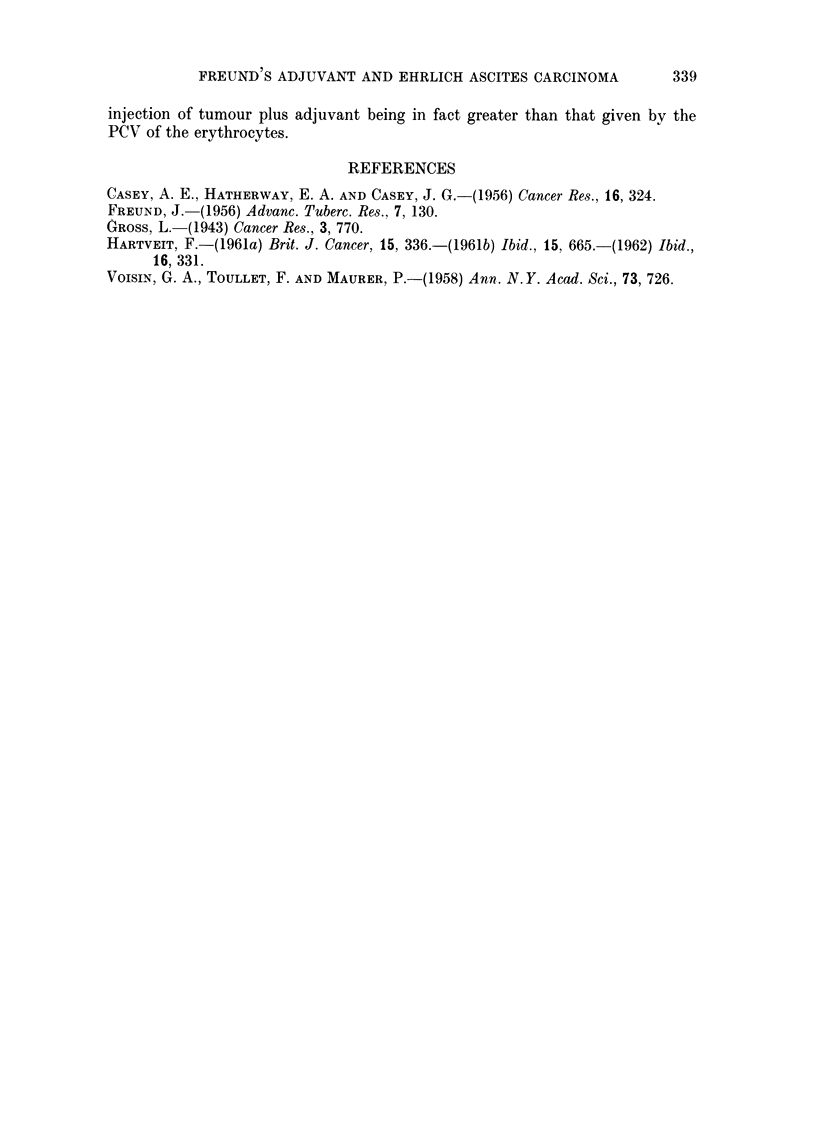# Further Observations on the Results of Combining Freund's Adjuvant with Living Ehrlich Ascites Carcinoma

**DOI:** 10.1038/bjc.1962.38

**Published:** 1962-06

**Authors:** F. Hartveit


### FURTHER OBSERVATIONS ON THE RESULTS OF COMBINING FREUND'S ADJUVANT WITH LIVING EHRLICH ASCITES CARCINOMA

F. HARTVEIT

*From the University of Bergen, School of Medicine, the Gade Insitute, Department of Pathology, Bergen, Norway*

Received for publication March 20, 1962

IN a previous paper (Hartveit, 1962) the production of immunity to Ehrlich's ascites carcinoma by means of the subcutaneous injection of the same tumour combined with Freund's adjuvant was studied. In the course of that experiment some further points arose. Firstly, to what extent did the combination with Freund's adjuvant damage the tumour cells? This will be reflected in the survival time of the mice and is discussed in part (a) of this paper. Secondly, a marked non-specific immune reaction was noticed in female mice following the subcutaneous injection of adjuvant alone. Would this reaction be influenced by a time interval between the subcutaneous and the intraperitoneal injection? This is discussed in part (b). Thirdly, it was noticed that haemolysis occurred in the tumour ascites in some of the mice. This was not mentioned previously and will now be presented in detail in part (c) of this paper together with the corresponding findings in part (b).

It will be noted that the experimental material concerned here is that used in the previous experiment (Harveit, 1962). The composition of the material and the treatment given are summarized in Table I, which also shows the additional treatment given to the mice in groups 1 and 4 in the experiment reported in part (b) of this paper.

TABLE I.—*The Composition of the Experimental Material and the Treatment given in the Different Groups.* (15 *Male and* 15 *Female Mice in Each Group*)

| Group | Route of injection: Subcutaneous (day 1) | Route of injection: Intraperitoneal |
|---|---|---|
| 1 | Nil | EAC (day 30) |
| 2 | Nil | EAC (day 1) |
| 3 | FA + EAC | Nil |
| 4 | FA + saline | EAC (day 30) |
| 5 | EAC + saline | Nil |
| 6 | FA +EAC | EAC (day 1) |
| 7 | FA + saline | EAC (day 1) |
| 8 | EAC + saline | EAC (day 1) |

FA = Freund's adjuvant. EAC = Ehrlich's ascites carcinoma.

The following experiments were carried out :

### (a) *The Survival Time of Mice following the Subcutaneous Injection of Ehrlich's Ascites Carcinoma and Freund's Adjuvant*

The two control groups with which we are concerned here are group 3, in which the mice were given a mixture of Freund's adjuvant and Ehrlich's ascites carcinoma subcutaneously, and its control group 5 in which they were given tumour alone (Table I). Comparison of the survival time in these two groups should establish the extent to which the tumour was injured by its combination with Freund's adjuvant.

*Material and methods*

For a detailed description of these the reader is referred to the original paper (Hartveit, 1962) and to Table I of this paper. To summarize: Each group contained 15 male and 15 female mice. The mice in group 3 were each given one subcutaneous injection of a mixture of Ehrlich's ascites carcinoma and Freund's adjuvant, while those in group 5 each received one subcutaneous injection containing the same number of tumour cells as those in group 3. The tumour came from the same source for both groups and, with the exception of the emulsifying process used before injection in group 3, was handled in the same way.

The mice were then examined weekly for the presence of subcutaneous tumour and any growth present was measured. The survival time was recorded in days.

*Results*

Tumour nodules occurred at the site of the subcutaneous injection in all animals. At one week there was little difference in the mean tumour diameter in groups 3 and 5 (9·7 mm. $\pm$ 2·1 and 9·2 mm. $\pm$ 3·9, respectively). By three weeks every tumour had reached at least 1 cm. in diameter (i.e. double the size of the nodules found on injection of adjuvant alone in females). Thereafter the tumours regressed in two animals in each group; in 2 females in group 3 and in 1 male and 1 female in group 5. These four mice were alive without sign of tumour one month after all the rest were dead. They were excluded from the survival time calculations. The remaining animals died with actively growing tumours.

Table II shows the mean survival time of these mice, with the SD of this mean, the SE of the difference between the female and the male means and its P value. It will be seen that the mean survival time of the animals of the same sex in both groups was approximately the same, and that the marked sex difference (0·001 > P) occurred in both groups.

TABLE II.—*The Mean Survival Time in Days* (*x*) *of Mice with Subcutaneous Ehrlich Ascites Carcinoma and Freund's Adjuvant* (*Group* 3) *Compared to that of Mice with Subcutaneous Ehrlich Ascites Carcinoma Alone* (*Group* 5), *Giving the Sex and Number of the Mice, the Mean Survival Time and SD, the SE of the Sex Difference in Means and its P value*

| Group | Sex | Number | $\bar{x}$ | S.D.$\bar{x}$ | S.E. ($\bar{x}$♀ − $\bar{x}$♂) | $P$ |
|---|---|---|---|---|---|---|
| 3 | ♂ | 15 | 48·5 | 16·4 | 4·74 | $0{\cdot}001 > P$ |
| | ♀ | 13*/* | 78·7 | 24·4 | | |
| 5 | ♂ | 14* | 49·3 | 13·45 | 4·48 | $0{\cdot}001 > P$ |
| | ♀ | 14* | 80·3 | 31·65 | | |

* One mouse survived, see text.

*Discussion*

The number of cells injured or killed during the emulsifying process undergone by the tumour cells in group 3 does not appear to have affected the survival time of the mice, which is almost identical to that of the controls which were given the same dose of untreated tumour. It may be, however, that a large majority of the cells were killed and that these dead cells exerted the well known XYZ effect (Casey, Hatherway and Casey, 1956) on those remaining. This could offset the reduced tumour cell dosage and bring the survival time back in line with that in group 5. In any case, the assumption made in the previous paper (Hartveit, 1962) that living Ehrlich ascites carcinoma was used in combination with Freund's adjuvant can be upheld.

The findings here show that the tumour cells in the adjuvant mixture continue to grow in the absence of a further source of tumour. In the previous experiment (Hartveit, 1962) it was found that the tumour cells in the same adjuvant mixture did not grow in the presence of intraperitoneal tumour. This rules out the prophylactic use of such a mixture while, at the same time, it indicates that the mixture may be of some use in the treatment of a tumour-bearing host.

It is of note that there was a marked sex difference in the survival time in both groups. This is contrary to the author's findings with interaperitoneal tumour (Hartveit, 1961*a*), and contrary to the general experience of the intraperitoneal growth of this tumour. It is likely that the rapid intraperitoneal growth rate prevents the sex difference, which becomes apparent following the slower subcutaneous growth, from showing up. Thus female mice, in which it is easier to induce tumour immunity (Gross, 1943), also have a greater natural immunity to the Ehrlich ascites carcinoma than male animals.

(b) *The Non-specific Action of Freund's Adjuvant on Ehrlich's Ascites Carcinoma*

In the course of the main experiment (Hartveit, 1962) it was found that female mice given a subcutaneous injection of Freund's adjuvant alone on the same day as an intraperitoneal injection of Ehrlich's ascites carcinoma showed a decrease in survival time and an increase in the blood content of their tumours when compared to male mice given the same treatment. This reaction must be considered to be non-specific in contrast to that produced by the subcutaneous injection of Freund's adjuvant plus living tumour. It is suggested in the above mentioned paper that the latter reaction is mediated through the same channels as that due to natural immunity, while the former clearly operates in a different way. It was also found that the female mice showed a marked reaction at the subcutaneous injection site while this was absent in the males.

The sex differences in the responses mentioned above were highly significant statistically. It was decided to follow up these findings to see if a time interval between the injection of the subcutaneous adjuvant and that of the intraperitoneal tumour would affect the reactions. A new experiment was, therefore, undertaken in which mice that had received Freund's adjuvant and saline subcutaneously were given intraperitoneal tumour 30 days later, instead of on the same day as in the main experiment (Hartveit, 1962).

*Material and methods*

The reader is referred to the previous paper (Hartveit, 1962) and to Table I of this paper for details. To summarize : There were 15 male and 15 female mice in each group. Group I was the untreated control group while group 4 received Freund's adjuvant subcutaneously. Thirty days after the mice in group 4 had been injected with adjuvant all the mice in both groups were given an intraperitoneal injection of 0·1 ml. of Ehrlich's ascites carcinoma (2,300,000 tumour cells/c.mm.). This tumour was taken from a mouse of the 87th transplant generation. The tumour used in the previous experiment had come from the 84th transplant generation of the same tumour.

At the time of the injection of the intraperitoneal tumour the subcutaneous swellings (approximately 0·5 cm. diameter), that had appeared within 14 days of the injection of the adjuvant mixture in the females, persisted in all cases. In the males the site was difficult to locate.

When the mice died the survival time was recorded in days and the blood content of the tumour recorded after centrifugation, as described in the previous paper (Hartveit, 1962). The subcutaneous injection site was examined at autopsy in group 4.

*Results*

Table III shows the mean survival time (days), plus the SD of the mean, of the mice in both groups. The female mice in the experimental group 4 showed little difference in survival time from those in the control group 1. On the other hand, there was a marked difference in the survival time of the male mice ; those in group 4 dying before the controls (13·64 and 17·4 days, respectively). This difference is statistically significant ($0{\cdot}05 > P > 0{\cdot}02$).

TABLE III.—*The Mean Survival Time (Days), plus SD, of Mice given Intraperitoneal Ehrlich's Ascites Carcinoma* 30 *days after Subcutaneous Freund's Adjuvant (Group* 1) *and of Controls lacking Subcutaneous Adjuvant (Group* 4)

| Group | Sex | Number of mice | Mean survival time | SD |
|---|---|---|---|---|
| 1 | ♂ | 15 | 17·4 | 6·332 |
| | ♀ | 15 | 12·3 | 3·194 |
| 4 | ♂ | 14* | 13·64 | 2·811 |
| | ♀ | 15 | 12·6 | 4·775 |

* One mouse accidentally killed.

Table IV gives the mean blood content of the tumour (per cent), plus the SD of the mean, in both groups. Once again there is little difference in the results in female mice, while the blood content in the males in the experimental group 4 is

TABLE IV.—*The Mean Blood Content (per cent), Plus SD, of the Intraperitoneal Tumour in Groups* 1 *and* 4

| Group | Sex | Number of mice | Mean blood content | SD |
|---|---|---|---|---|
| 1 | ♂ | 14† | 3·143 | 2·530 |
| | ♀ | 15 | 5·2 | 4·651 |
| 4 | ♂ | 13*† | 5·0 | 2·037 |
| | ♀ | 15 | 5·266 | 3·347 |

* One mouse accidentally killed.
† Tumour unsuitable for investigation in one mouse.

much higher than in the controls (5 and 3·14 per cent, respectively). This difference is significant statistically (0·05>P>0·2).

Table V shows the correlation between the survival time and the tumour blood content (correlation coefficient, r) for both sexes in both groups. While the negative correlation is high in the females in both groups, it is much lower in the males in group 4 than in group 1.

TABLE V.—*The Correlation between Survival Time and Tumour Blood Content in Groups* 1 *and* 4, *using the Correlation Coefficient r*

| Group | Sex | Number of mice | *r* |
|---|---|---|---|
| 1 | ♂ | 14† | −0·5163 |
| | ♀ | 15 | −0·8501 |
| 4 | ♂ | 13*† | −0·1642 |
| | ♀ | 15 | −0·8027 |

* One mouse accidentally killed.
† Tumour unsuitable for investigation in one mouse.

At autopsy the subcutaneous injection site was easily located in 14 out of the 15 females as the subcutaneous swelling had persisted. It could not be detected macroscopically in the males.

*Discussion*

It has previously been shown that the survival time of mice with intraperitoneal Ehrlich ascites carcinoma is shorter in those that show a sensitivity reaction to the tumour, and that the blood content of the tumour ascites is a measure of this reaction, which is probably one of rejection of the foreign tumour protein (Hartveit, 1961*b*). Thus it provides a measure of the animal's natural immunity to the tumour. In addition there is also evidence that it can be used to measure acquired immunity (Hartveit, 1962).

In the latter experiment it was found that female mice showed this immunity reaction if they were given a subcutaneous injection of Freund's adjuvant without antigen on the same day as the intraperitoneal injection of Ehrlich's ascites carcinoma. This was unexpected as Freund (1956) clearly states that, " the adjuvant remains without effect if injected into a separate area ", i.e. if antigen is not combined with adjuvant. On the other hand, Voisin, Toullet and Maurer (1958) have reported non-specific testicular lesions following the use of adjuvant alone.

In the present experiment the adjuvant that was given subcutaneously in group 4 was the same as that used in group 7 of the main experiment (i.e. the mice that received subcutaneous adjuvant on the same day as they were given intraperitoneal tumour). The tumour used was taken from the same source 3 transplant generations later. Thus conditions in the two experiments are strictly comparable—the time limit between the subcutaneous and intraperitoneal injections being the determining factor.

The results show that it is now the male animals that are responding to the adjuvant injection—the females reacting in the same way as the controls. The mean survival time of the males is now significantly less than that of the control males (13·64 days compared to 17·4 days), and the blood content of their tumours greater (5 per cent compared to 3·14 per cent). In addition the degree of correlation between the survival time and the tumour blood content is greatly reduced (−0·1642 compared to −0·5163). The subcutaneous injection sites did not show any change.

The mean survival time of the females, the blood content of their tumours and the degree of correlation between these two factors now appears to be unchanged by the treatment.

These results show that the time interval in this case was important to the response of the mice. The immune reaction of the females developed shortly after the injection of the adjuvant, while that of the males became evident later. In the main paper (Hartveit, 1962) it was suggested that the reaction in the females differed in mechanism from that due to natural immunity or immunity acquired after the subcutaneous injection of Freund's adjuvant and living Ehrlich ascites carcinoma, as the negative correlation between the survival time and the tumour blood content, that is normally found in these mice, was upset by the treatment. The present results support this idea as once more the animals responding to the subcutaneous injection of adjuvant alone (now only the males) show the same lack of correlation between these two factors. The experiment also shows that the immune reaction is not dependent on the reaction at the subcutaneous injection site.

### (c) *Haemolysis in the Ehrlich Ascites Carcinoma following the Subcutaneous Injection of Whole Tumour Fluid plus Freund's Adjuvant*

In the course of the analysis of the results of the main experiment (Hartveit, 1962) and of part (b) of this paper, it came to light that there were marked differences in the amount of haemolysis in the ascitic fluid in the various groups. These findings will now be presented in detail.

#### *Material and methods*

For a full description of these the reader is referred to the original paper (Hartveit, 1962), and to part (b) and Table I of this paper. To summarize : All groups contained 15 male and 15 female mice. The experimental group 6 was given a mixture of Freund's adjuvant and living Ehrlich ascites carcinoma subcutaneously, while groups 1 and 2 had no subcutaneous treatment. Groups 4 and 7 were given subcutaneous adjuvant minus tumour ; group 8 subcutaneous tumour alone. On the first day of the experiment the mice in groups 2, 6, 7 and 8 each received intraperitoneal Ehrlich ascites carcinoma. Thirty days later groups 1 and 4 were given intraperitoneal tumour. The remaining groups are not concerned in this experiment.

When the mice died the tumour ascites was removed and centrifuged as described previously. After centrifugation the haemolysis in the supernatant fluid was graded :

0 = absent, pale yellow supernate.
+ = present, pink supernate.
++ = marked, dark red supernate.

*Results*

The findings in both the males and the females are given in Table VI which shows that ++ haemolysis occurred only in groups 6, 7 and 4 (in 30 per cent, 3·3 per cent and 3·6 per cent respectively). The difference in this percentage between group 6 and the untreated mice (group 2) is highly significant statistically ($0 \cdot 01 > P > 0 \cdot 001$). There were no marked sex differences.

TABLE VI.—*Haemolysis in the Ascitic Fluid of Mice with Intraperitoneal Ehrlich Ascites Carcinoma (EAC) following the Subcutaneous Injection of Living EAC plus Freund's Adjuvant (FA), and in Control Groups.* (30 *Mice in Each Group*)

| Group | Treatment | Sex | Haemolysis+ 0 | + | ++ | %++ |
|---|---|---|---|---|---|---|
| 2 | Nil | ♂ | 10 | 5 | 0 | 0 |
| | | ♀ | 10 | 5 | 0 | |
| 6 | EAC + FA | ♂ | 8 | 3 | 4 | 30 |
| | | ♀ | 8 | 2 | 5 | |
| 7 | FA | ♂ | 12 | 3 | 0 | 3·3 |
| | | ♀ | 11 | 3 | 1 | |
| 8 | EAC | ♂ | 12 | 3 | 0 | 0 |
| | | ♀ | 11 | 4 | 0 | |
| 1‡ | Nil | ♂† | 11 | 3 | 0 | 0 |
| | | ♀ | 12 | 3 | 0 | |
| 4‡ | FA | ♂*† | 11 | 2 | 0 | 3·6 |
| | | ♀ | 12 | 2 | 1 | |

* One mouse accidentally killed.
† Tumour unsuitable for investigation in one mouse.
+ For details of scale see text.
‡ For details see part b.

*Discussion*

It was decided to ignore + haemolysis as the time that elapsed between the death of the mouse and the centrifugation of the tumour ascites could not be determined accurately ; differences of up to 18 hr. might have occurred. It was thought, however, that gross changes, i.e. ++ haemolysis, were unlikely to have been brought about in this way—and were as likely to occur in all groups concerned on this basis.

It was found that this degree of haemolysis was virtually confined to mice in group 6, that is to say to the mice that had been given tumour plus adjuvant subcutaneously. The tumour used for injection consisted of whole ascitic fluid. No attempt was made to remove the erythrocytes, which gave a PCV of 1 per cent Wintrobe, as such a procedure might have been detrimental to the tumour cells. Therefore the tumour adjuvant mixture also contained erythrocytes.

The results of the experiment suggest that the combination of erythrocyte-adjuvant may have been the cause of the increased haemolysis in the experimental group. It did not occur following the administration of whole ascitic fluid alone, i.e. tumour, erythrocytes and ascitic serum (group 8), and there was only a very minor non-specific reaction following subcutaneous adjuvant alone. It was also absent in the untreated controls.

Considering these findings in relation to those in the main experiment (Hartveit, 1962) it could be argued that it was this haemolysis that was detrimental to the mice in the experimental group, and that it was this, and not a reaction against the tumour protein, that played a major part in their early death. If only the survival time is considered this appears quite possible, but when taken in conjunction with the tumour blood content it is evident that this is unlikely. While one could well expect treatment with an erythrocyte-adjuvant mixture to increase the haemolysis of the blood present in the tumour, one could hardly expect that to account for an increase in the blood content itself.

Secondly it could be argued that group 6 showed the greatest amount of haemolysis as the tumours contained the greatest amounts of blood. However, on referring back to Table III of the original paper (Hartveit, 1962) it will be seen that the females in group 7 showed almost as high a tumour blood content as those in group 6, while they do not show a correspondingly high degree of haemolysis.

Thus the haemolysis will have detracted from the findings on the blood content of the tumour ascites in group 6 as judged from the packed cell volume of the erythrocytes present. The amount of blood present was, in fact, even greater than that recorded in this group. It is of note that this reaction to a normal tissue did not show a sex difference while that related to the tumour tissue did.

### SUMMARY

(a) Emulsification of Ehrlich's ascites carcinoma with Freund's adjuvant does not appear to have adversely effected the growth of the tumour cells in the mixture when this is injected subcutaneously into healthy mice. On subcutaneous injection of the tumour the female mice show greater natural immunity than the males as evidenced by their longer survival time.

(b) A non-specific immune type reaction to Ehrlich's ascites carcinoma was found in male mice following the subcutaneous injection of Freund's adjuvant mixture 30 days before the intraperitoneal injection of Ehrlich's ascites carcinoma. Female mice showed no reaction after this time interval, and the time interval had no effect on the reaction at the site of the subcutaneous adjuvant injection. Further the immune reaction was not dependent on the latter. While the mechanism of the immune reaction is thought to be similar to that occurring in female mice when the adjuvant is given on the same day as the tumour, it is suggested that it differs from that accompanying natural immunity and from the immunity acquired after the subcutaneous injection of Freund's adjuvant plus living tumour.

(c) It was found that haemolysis occurred in intraperitoneal Ehrlich ascites carcinoma following the subcutaneous injection of whole Ehrlich ascites carcinoma plus Freund's adjuvant. Marked haemolysis was absent in the untreated control groups. This haemolysis is thought to have been due to the erythrocytes present in the ascitic tumour fluid that was combined with the adjuvant mixture. The haemolysis will have detracted from the findings in the main experiment ; the haemorrhagic response to the intraperitoneal tumour following the subcutaneous injection of tumour plus adjuvant being in fact greater than that given by the PCV of the erythrocytes.

### REFERENCES

Casey, A. E., Hatherway, E. A. and Casey, J. G.—(1956) *Cancer Res.*, **16**, 324.
Freund, J.—(1956) *Advanc. Tuberc. Res.*, **7**, 130.
Gross, L.—(1943) *Cancer Res.*, **3**, 770.
Hartveit, F.—(1961*a*) *Brit. J. Cancer*, **15**, 336.—(1961*b*) *Ibid.*, **15**, 665.—(1962) *Ibid.*, **16**, 331.
Voisin, G. A., Toullet, F. and Maurer, P.—(1958) *Ann. N.Y. Acad. Sci.*, **73**, 726.